# Predictors of COVID-19 Preventive Behavior Adoption Intention in Malaysia

**DOI:** 10.3389/fpsyg.2021.616749

**Published:** 2021-05-20

**Authors:** Norazryana Mat Dawi, Hamidreza Namazi, Petra Maresova

**Affiliations:** ^1^Sunway University Business School, Sunway University, Selangor, Malaysia; ^2^Center for Basic and Applied Research, Faculty of Informatics and Management, University of Hradec Kralove, Hradec Kralove, Czechia; ^3^School of Engineering, Monash University, Selangor, Malaysia; ^4^Department of Economics, Faculty of Informatics and Management, University of Hradec Kralove, Hradec Kralove, Czechia

**Keywords:** COVID-19, e-government, social media, theory of reasoned action, preventive behavior

## Abstract

Preventive behavior adoption is the key to reduce the possibility of getting COVID-19 infection. This paper aims to examine the determinants of intention to adopt preventive behavior by incorporating perception of e-government information and services and perception of social media into the theory of reasoned action. A cross-sectional online survey was carried out among Malaysian residents. Four hundred four valid responses were obtained and used for data analysis. A partial least-square-based path analysis revealed direct effects of attitude and subjective norm in predicting intention to adopt preventive behavior. In addition, perception of e-government information and services and perception of social media were found to be significant predictors of attitude toward preventive behavior. The findings highlight the importance of digital platforms in improving people’s attitudes toward preventive behavior and in turn contain the spread of the infectious disease.

## Introduction

The novel coronavirus (COVID-19) pandemic has affected millions of lives around the world. This is a challenging time for humanity, and everybody must work together to combat the disease, as it can be easily transmitted between humans. During the period of 10 months since the first case in Wuhan, China was identified, the virus has killed nearly 1 million of the world’s population. In terms of economic impact, the COVID-19 pandemic has resulted in economic shutdowns, specifically in primary sectors such as the extraction of raw materials, secondary sectors such as the production of end products, and tertiary sectors such as service industries (Nicola et al., 2020). The detrimental effects brought by the virus have urged the public health authorities to improve preventive behavior strategies in containing the outbreak continuously. The use of e-government and social media during the pandemic suggests a means to disseminate information and educate people on public health recommendations.

In the history of modern public health, several respiratory disease outbreaks had happened, such as H1N1, severe acute respiratory syndrome (SARS), and Middle East respiratory syndrome coronavirus (MERS-CoV), but quick actions were able to be applied to contain the spread of the viruses. In the case of the COVID-19 pandemic, up to the date of writing, no effective vaccine or medicine has been found to fight the virus (Mal et al., 2020). Governments around the world are reliant on non-pharmaceutical interventions (NPIs) such as social distancing and washing hands regularly to contain the virus transmissions (Doogan et al., 2020). According to Bhatia (2020), there is a need to empower and educate the public to practice NPIs since pharmaceutical interventions are not within reach to developing countries in the near future. Furthermore, people are becoming tired of the situation and start to ignore health recommendations (Parrish, 2020). Therefore, it is important to study how to influence people to engage in preventive behavior.

There is a substantial body of research concerning the public behavior on prevention and control of COVID-19 transmission. Several authors examined the impact of government proactive actions in handling the outbreak (Ahmad et al., 2020; Al-Hasan et al., 2020; Dai et al., 2020). Other researchers examined the effects of social media use for information dissemination during the COVID-19 outbreak (Allington et al., 2020; La et al., 2020). Some studies have focused on individual factors such as attitude toward the effectiveness of epidemic preventive, conscientiousness, neuroticism, and personal hygiene practices on preventive behavior adherence (Abdelrahman, 2020; Chen and Chen, 2020).

Despite the numerous interests of the previous research on preventive behavior during COVID-19, several gaps remain for research in this area. Studies on people’s perception of electronic government (hereafter e-government) adoption during a health crisis are scarce. E-government has been widely used during the COVID-19 pandemic as a means to broadcast information and service delivery to the citizens (United Nations [UN], 2020). Due to restrictions such as controlled movement and social distancing, the use of information and communication technologies (ICTs) has helped the government to reach the public, especially to disseminate emergency information and to provide essential services. Providing citizens with accurate information during a pandemic is important to raise the public’s risk awareness and lead people to perform preventive behavior (Paek et al., 2008). In addition, the interactivity of digital platforms reinforces trust between the government and citizens (Parent et al., 2005), which may influence people to be more engage in governmental practices. Thus, a question was raised on how people’s perception of information and services provided by e-government during the COVID-19 pandemic affect their attitude toward preventive behavior.

Social media is another important online platform during the COVID-19 outbreak used for information creation, dissemination, and consumption. A study by Lin et al. (2020) figured out that people use social media during the COVID-19 outbreak to obtain information related to preventive behavior, virus transmission, and disease symptoms. There is a rising body of social media literature examining health environments, such as public health surveillance, promotion, and communication (Giustini et al., 2018; Jenkins et al., 2020). Studies related to the roles of social media on public health during the COVID-19 pandemic have focused on areas such as public attitudes, assessing mental health, and evaluation of prevention education (Tsao et al., 2021). However, there is limited knowledge on how people’s perception of social media during the COVID-19 pandemic affect attitude toward preventive behavior. Thus, it is worth studying this area to enrich the literature.

Previous studies also have neglected the influence of sociability in affecting preventive behavior adoption during the COVID-19 pandemic. During a health crisis, it is important to communicate with family and friends to ensure everyone is safe. Thus, a question was raised about whether close acquaintances influence a person’s intention to adopt preventive behavior. As stated by Bagozzi and Lee (2002), social factors are important in an individual’s decision-making process. People’s decision to perform preventive behavior during the COVID-19 outbreak may be influenced by the people that are significant to them. As people’s behavior is associated with society, empirical research is needed to test it.

This study attempts to examine the intention to adopt preventive behavior in terms of the drivers in Malaysia. A behavioral framework based on the theory of reasoned action (TRA) is modified to incorporate perception of e-government information and services and perception of social media in determining preventive behavior adoption. The findings of this paper may help academics and policymakers to gain insights on the improvement of preventive behavior adoption during the COVID-19 pandemic.

The remainder of this paper is structured as follows: section “Theoretical Framework and Hypotheses Development” explains the theoretical framework and hypotheses development. Section “Materials and Methods” details the methodology of the research. Section “Results” demonstrates the results. Section “Discussions” discusses the findings. Lastly, section “Conclusion” concludes the research.

### Theoretical Framework and Hypotheses Development

The TRA by Ajzen and Fishbein (1980) has been tested in multiple health-related behaviors such as vaccination (Kan and Zhang, 2018), physical activity (Sarbazi et al., 2019), and healthy eating (Kamar et al., 2016). According to the theory, an individual’s engagement in a specific behavior is being determined by the attitude toward the behavior, subjective norm, and behavioral intention (Fishbein and Ajzen, 1975). TRA posits that actual behavior is predicted by the intention to perform that behavior, while the intention is influenced by subjective norm and attitude. Subjective norm is the social pressure perceived by an individual in performing a behavior, while attitude refers to a person’s belief about the outcome of performing a behavior. Applying TRA to the research context of this study, individuals’ perception of e-government information and services and perception of social media during COVID-19 pandemic were believed to form their attitudes toward preventive behavior, which then predicted their intention and finally performing the actual preventive behavior against the virus.

### E-Government During the COVID-19 Pandemic

E-Government is defined as the use of ICTs, which enable relational transformation between people, businesses, and governmental bodies (The World Bank [TWB], 2019). E-government promotes efficiency and effectiveness in governance, improves the accessibility of government services and information, and helps the government to be more accountable to the citizen (Yang and Rho, 2007). The use of e-government has been escalated during the COVID-19 pandemic. For instance, in Singapore and Pakistan, the governments collaborate with telecommunication companies to develop mobile applications for contact tracing purposes. The government of Canada has established an online self-assessment website to help citizens determine whether they need to be tested for COVID-19. In the United Kingdom, 69 new digital services were designed and implemented during the COVID-19 crisis including a website to guide vulnerable people to get help such as the delivery of basic supplies and a portal to facilitate people in applying COVID-19 economic stimulus packages.

In Malaysia, the government adopts several e-government practices during the COVID-19 pandemic. These involve the use of numerous digital platforms such as websites and portals, social media, and mobile applications. The Ministry of Health Malaysia (MOH) has developed a portal^1^ to provide information related to COVID-19 such as the number of infected cases and COVID-19 guidelines. Mobile applications called “MySejahtera” and “MyTrace” were developed using QR code log systems for contact tracing purposes. Using these applications, people need to scan a unique QR code when entering places such as shopping stores or restaurants to register their visits. If someone that visited the place gets infected by the virus, the information will be promptly disseminated to other visitors, and appropriate measures could be adopted. MOH also used their Facebook page to provide up-to-date information and to live broadcast the daily press conference by the Director-General of Health. In addition, an operational system called eCOVID19, which comprises analytical tools and various dashboards, was developed specifically to help the government in decision making and monitoring the situation. Other than managing the spread of the virus, electronic systems also are used to ensure that the public continues receiving government services during the pandemic. For instance, an electronic appointments system was developed to facilitate departments such as immigration, tax, and healthcare. Besides, a portal called “MySafeTravel” provides travelers coming into the country to conduct COVID-19 screening and quarantine accommodation arrangements. Clearly, the government of Malaysia leverages e-government platforms during the pandemic. In fact, a recent study by Bertrand and McQueen (2021) figured out that 88% of Malaysian citizens said the government had effectively used digital technology to respond to the pandemic.

Undeniable, government plays an important role during a health crisis especially for risk communication to ensure that citizens are well-informed about the condition. According to Yusof et al. (2020), apart from reducing anxiety and panic, effective risk communication during the COVID-19 pandemic will educate the public to adopt necessary preventive behavior. Tursunbayeva et al. (2017) suggested that the application of e-government in public health could facilitate transparency and accountability of public services and attract public engagement. To some extent, people’s attitudes toward adopting preventive behavior are relied upon how they perceive the government commitment in handling the situation. Positioning this in the context of e-government adoption, people’s perception of the information and services provided by e-government during the COVID-19 pandemic are expected to influence their attitudes toward preventive behavior. The more positive individuals perceived the information and services provided by e-government, the more positive their attitudes toward adopting preventive behavior.

**Hypothesis 1 (H1):** Perception of e-government information and services is positively related to attitude toward preventive behavior during the COVID-19 pandemic.

### Social Media During the COVID-19 Pandemic

Social media has become the primary communication medium during the COVID-19 pandemic (Kaya, 2020; Bekele et al., 2021; Vatan et al., 2021). The rapid global spread of coronavirus has increased the use of social media, which may give a significant impact on the users. People use social media as the platform for public opinions, perceptions, and attitude toward the multiple events occurred during the pandemic. A recent report that analyzed social media consumption found that 36% of people visit social media to stay up to date with the current news and events related to COVID-19 (Trifonova, 2020). People tend to share similar feelings during unprecedented times in social media and converse about it to get more information.

In Malaysia, social media is used by Malaysians during the COVID-19 pandemic as the platform to show their sense of togetherness through the proliferation of positive messages (Azizan et al., 2020). Social media has become the place to brighten up each other’s spirit. For instance, people share their patriotic spirits by complimenting the government and frontliners’ efforts in combating the pandemic. People also use social media to share their supports for creative and beneficial activities performed by others during lockdowns such as cooking skills and charity works for vulnerable people. Besides, hashtags and phrases such as “stay at home” and “*kita jaga kita*,” which means “we take care of each other” are constantly shared among social media users in Malaysia to show their solidarity with each other during the difficult time. Social media users also tend to share positive tips and advice on how to protect themselves from getting infected by the virus. The ability to share news and information between the users makes social media the preferred medium for Malaysians to seek the latest updates related to the COVID-19 outbreak.

Even though the use of social media during the COVID-19 crisis is being linked to misinformation (Cinelli et al., 2020; Chou et al., 2021; Fernández-Torres et al., 2021), social media could play a positive role if the information disseminated is clear and accurate (Hauer and Sood, 2020). Social media use during a crisis is said to be an effective way of knowledge management, coordinate important information from different sources, and promote situational awareness (Hughes and Palen, 2009; Yates and Paquette, 2011; Cui and Kertész, 2021). Al-Dmour et al. (2020) found out that the use of social media platforms such as Facebook and Twitter helped people to recognize COVID-19 and directly influence public health protection. In Malaysia, Zhang S. X. et al. (2020) figured out that the more time people spent on social media is linked with more handwashing behavior to protect themselves from the virus. We argue that the positive effect of social media on health behavior depends on an individual’s perception of the platform. It is expected that people’s perception of social media influences their attitude toward preventive behavior. The more positive they perceive social media, the more positive their attitudes toward adopting preventive behavior.

**Hypothesis 2 (H2):** Perception of social media is positively related to attitude toward preventive behavior during the COVID-19 pandemic.

### Subjective Norm

As theorized in TRA, the subjective norm is a predictor of behavioral intention. It reflects the extent to which individuals perceived people that are significant to them think they should perform or not perform a specific behavior (Ajzen and Fishbein, 1980). The pressure to perform a particular behavior is increased if there is a reward or punishment given by the social actors (Fishbein and Ajzen, 1975). In the context of COVID-19, protecting family members from getting infected by the virus is important, as people who are living within the household are at high risk of transmitting the virus (Li et al., 2020). Therefore, engaging in preventive behavior not only helps individuals to protect themselves but also reduce contagion risks of their loved ones. Qazi et al. (2020) found out that informal information from family and friends affect people’s awareness of health preventive behavior during the COVID-19 pandemic. This shows the importance of close acquaintance in influencing individuals’ intention to adopt preventive behavior. In this study, the subjective norm is defined as the degree to which an individual perceives that others think he/she should adopt preventive behavior against COVID-19. Therefore, we hypothesized that subjective norm positively affects intention to adopt preventive behavior.

**Hypothesis 3 (H3):** Subjective norm is positively related to intention to adopt preventive behavior during COVID-19 pandemic.

### Attitude Toward Preventive Behavior

Attitude is an important variable in TRA due to its power and capability to predict behaviors (Kraus, 1995). According to TRA, attitude toward behavior refers to the general overall affective feeling of a person’s favorability for the behavior. It is the function of perceived outcomes of performing the specific behavior and the subjective evaluation of the outcomes (Fishbein and Ajzen, 1975). In this study, we defined attitude toward preventive behavior as the degree to which a person’s attitude is favorable or unfavorable toward preventive behavior adoption. Wang et al. (2021) suggest that individual attitudes toward compliance behavior directly affect public health efforts during the pandemic. Attitude has long been recognized as the antecedent of behavioral intention (Suki and Suki, 2011). Therefore, we hypothesized that attitude toward preventive behavior positively affects intention to adopt preventive behavior.

**Hypothesis 4 (H4):** Attitude toward preventive behavior is positively related to intention to adopt preventive behavior during the COVID-19 pandemic.

This study emphasizes on the impact mechanism and path through which perception of e-government information and services and perception of social media affect an individual’s attitude toward preventive behavior. Furthermore, we also consider exploring the path through which perception of e-government information and services and perception of social media affect intention through mediating variable of attitude. It is important to study the mediating effect of attitude to provide a better understanding and to add a new contribution to the area of study (Kaakeh et al., 2019). Gilchrist et al. (2019); Laguía et al. (2019), and Mehmood et al. (2018) found that attitude mediates the relation between intention and other variables. Attitude has been tested as a mediating variable in the field of health behavior (Ebrahim et al., 2016; Zhang Y. et al., 2020). The current study tries to test this idea in the preventive behavior adoption field. It is expected that the relationship between perception of e-government information and services and perception of social media with intention to adopt preventive behavior could be mediated by the attitude toward preventive behavior. Hence, the following hypotheses were established:

**Hypothesis 5 (H5):** Attitude toward preventive behavior mediates the relationship between perception of e-government information and services and intention to adopt preventive behavior during the COVID-19 pandemic.

**Hypothesis 6 (H6):** Attitude toward preventive behavior mediates the relationship between perception of social media and intention to adopt preventive behavior during the COVID-19 pandemic.

**FIGURE 1:**
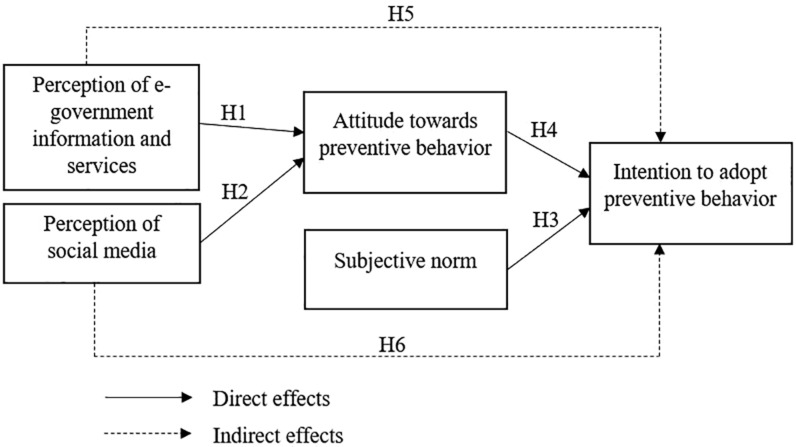
Theoretical model.

Based on the above theoretical conception, we examined intention to adopt preventive behavior predicted by subjective norm, attitude toward preventive behavior, perception of e-government information and services, and perception of social media. A hypothesized path model was proposed to test the direct and indirect effects between the variables, as illustrated in Figure 1.

## Materials and Methods

### Participants and Data Collection

An online survey was conducted during the Recovery Movement Control Order in Malaysia (RMCO) between June 7 and July 16, 2020. The respondents consisted of Malaysian residents and were recruited using a convenience sampling procedure. We obtained 404 usable responses that were used for the data analysis. Table 1 shows the detailed demographic of the sample.

**TABLE 1:** The demographic information of the respondents.

		Frequency	Percent	Cumulative Percent
Gender	Male	141	34.9	34.9
	Female	263	65.1	100.0
Age	18–24	336	83.2	83.2
	25–34	29	7.2	90.3
	35–44	24	5.9	96.3
	45–54	12	3.0	99.3
	55–64	3	0.7	100.0
Marital status	Single	351	86.9	86.9
	Married	49	12.1	99.0
	Divorced	4	1.0	100.0
Race	Malay	75	18.6	18.6
	Chinese	228	56.4	75.0
	Indian	35	8.7	83.7
	Other	66	16.3	100.0
Education	Ph.D.	11	2.7	2.7
	Master’s degree	19	4.7	7.4
	Bachelor’s degree	207	51.2	58.7
	Diploma	32	7.9	66.6
	A-Level	121	30.0	96.5
	Primary/Secondary School	14	3.5	100.0
Residence area	Urban	269	66.6	66.6
	Suburb	112	27.7	94.3
	Rural	23	5.7	100.0

### Measurement Instrument

Perception of e-government information and services was measured by seven items including the perceived quality of the information, the quality of services provided, and trustworthiness, reliability, and competence of the information provided over e-government channels and platforms. Perception of social media was assessed by nine items including evaluation of the information from social media, perceived effects of the information from social media, and perceived impact of social media platform. Five items of attitude toward preventive behavior, six items of subjective norm, and three items of intention to engage in preventive behavior were adapted from Ajzen and Fishbein (1980). All of the items were measured using a 5-point Likert scale ranging from 1 (strongly disagree) to 5 (strongly agree). The items were reviewed by four experts in the field of public health and information systems. Necessary changes were made based on their feedback and suggestions to establish face and content validity of the instrument. Ethical approval was obtained from Monash University Human Research Ethics Committee (Project ID: 24906).

### Data Analysis

The research aimed to explore the causal relationships between perception of e-government information and services, perception of social media, attitude toward preventive behavior, subjective norm, and intention to adopt in preventive behavior. This study also examined the mediating role of attitude toward preventive behavior. The partial least structural equation model (PLS-SEM) was used to test the research model using SmartPLS version 3 software. A two-stage procedure was involved in analyzing the data (Hair et al., 2017). First, the measurement model was analyzed by testing the reliability and validity of the constructs. Second, the structural model was examined to test the relationships among the constructs.

## Results

### Measurement Model

The reliability and validity of the model were assessed, and the results are shown in Table 2. Individual item reliability was examined based on outer loading value. All of the items surpassed the recommended value of 0.50 (Hair et al., 2017). Cronbach’s alpha and composite reliability were used to assess the constructs’ reliability with a cutoff value of 0.70 (Fornell and Larcker, 1981). The Cronbach’s alpha values ranged between 0.878 and 0.929, and composite reliability values ranged between 0.906 and 0.955, indicating good constructs reliability. The average variance extracted (AVE) was used to assess the validity of the constructs with values ranging between 0.541 and 0.876, exceeding the recommended value of 0.50 (Fornell and Larcker, 1981).

**TABLE 2:** Reliability and validity of measurement scales.

Constructs	Items	Outer loading	Cronbach’s alpha	Composite reliability	AVE
EGOVT	EGOVT1: E-government provides satisfactory quality of COVID-19 information.	0.757	0.878	0.906	0.581
	EGOVT 2: E-government services offered during the COVID-19 outbreak are satisfactory.	0.671			
	EGOVT 3: The government shows its commitment to curb the COVID-19 outbreak through e-government.	0.684			
	EGOVT 4: E-government is a trustworthy source to provide COVID-19 information.	0.798			
	EGOVT 5: COVID-19 information acquired from e-government is competent.	0.839			
	EGOVT 6: E-government provides reliable information on COVID-19.	0.826			
	EGOVT 7: I depend on e-government to obtain COVID-19 information.	0.741			
SOCMED	SOCMED1: I consider opinions from social media while selecting COVID-19 information.	0.551	0.894	0.913	0.541
	SOCMED2: Social media is a good source to get information on COVID-19 preventive behavior.	0.765			
	SOCMED3: I can change my opinion about COVID-19 based on updates reported on social media.	0.648			
	SOCMED4: Social media educates me on COVID-19 outbreak procedures.	0.821			
	SOCMED5: Social media educates me on preventive behavior to control COVID-19 infection.	0.846			
	SOCMED6: Social media spreads COVID-19 awareness in the community.	0.770			
	SOCMED7: Social media educates people on how to protect others if they are ill.	0.816			
	SOCMED8: Social media decreases people’s fear, anxiety, and confusion about COVID-19.	0.656			
	SOCMED9: Social media is a trustworthy source to provide COVID-19 information.	0.693			
SUBNORM	SUBNORM1: People who are important to me think I should comply with preventive behavior.	0.889	0.888	0.916	0.653
	SUBNORM2: People who are important to me think it is a good idea for me to comply with preventive behavior.	0.901			
	SUBNORM3: People who are important to me want me to comply with preventive behavior.	0.893			
	SUBNORM4: People who are important to me expect me to comply with preventive behavior.	0.864			
	SUBNORM5: The opinion of my family and friends about COVID-19 preventive behavior are important for me.	0.692			
	SUBNORM6: I follow the opinion of people who are important to me about COVID-19 preventive behavior.	0.541			
ATT	ATT1: I believe that engaging in preventive behavior will help me to avoid COVID-19 infection.	0.874	0.897	0.924	0.709
	ATT2: I believe that engaging in preventive behavior will help me to avoid COVID-19 infection.	0.839			
	ATT3: I believe that engaging in COVID-19 preventive behavior will help to maintain my health.	0.870			
	ATT4: I feel healthy when I engage in COVID-19 preventive behavior.	0.829			
	ATT5: I feel good about myself when I engage in COVID-19 preventive behavior.	0.796			
INT	INT1: I expect myself to engage in COVID-19 preventive behavior.	0.939	0.929	0.955	0.876
	INT2: I want to engage in the COVID-19 preventive behavior.	0.922			
	INT3: I intend to engage in COVID-19 preventive behavior	0.948			

*EGOVT, perception of e-government information and services; SOCMED, perception of social media; SUBNORM, subjective norm; ATT, attitude toward preventive behavior; INT, intention to adopt preventive behavior; AVE, average variance extracted.*

Additionally, discriminant validity was assessed by the Fornell–Larcker criterion and heterotrait–monotrait ratio (HTMT). As shown in Table 3, the discriminant validity was confirmed, as the square roots of the AVEs are larger than the correlations between constructs. Following the recommendation by Hair et al. (2017), the HTMT values were lower than the threshold of 0.85, indicating good discriminant validity.

**TABLE 3:** Discriminant validity.

Fornell–Larcker criterion					
	ATT	EGOVT	INT	SOCMED	SUBNORM
ATT	0.842				
EGOVT	0.405	0.762			
INT	0.570	0.369	0.936		
SOCMED	0.303	0.407	0.309	0.735	
SUBNORM	0.450	0.353	0.587	0.291	0.808

**Heterotrait–monotrait ratio (HTMT)**					

	**ATT**	**EGOVT**	**INT**	**SOCMED**	**SUBNORM**

ATT					
EGOVT	0.451				
INT	0.622	0.406			
SOCMED	0.307	0.451	0.319		
SUBNORM	0.504	0.411	0.637	0.339	

*EGOVT, perception of e-government information and services; SOCMED, perception of social media; SUBNORM, subjective norm; ATT, attitude toward preventive behavior; INT, intention to adopt preventive behavior.*

### Structural Model

A bootstrapping procedure (5,000 resamples) was conducted to examine the structural model in terms of its path significance based on the generated *t* and *p*-values. Table 4 presents the results of direct and indirect effects along with the overall fit statistics.

**TABLE 4:** Structured model.

Direct effect	Relation	β	*t*-value	*p*	Decision
**Hypotheses**					
H1	EGOVT→ATT	0.338	6.849	<0.001**	Supported
H2	SOCMED→ATT	0.165	3.478	0.001*	Supported
H3	SUBNORM→INT	0.414	7.887	<0.001**	Supported
H4	ATT→INT	0.383	7.050	<0.001**	Supported
**Specific indirect effect**	**Mediation path**				
H5	EGOVT→ATT→INT	0.130	4.558	< 0.001**	Full mediation
H6	SOCMED→ATT→INT	0.064	2.890	0.004*	Full mediation

**p < 0.01; *p < 0.05. EGOVT, perception of e-government information and services; SOCMED, perception of social media; SUBNORM, subjective norm; ATT, attitude toward preventive behavior; INT, intention to adopt preventive behavior.

**FIGURE 2:**
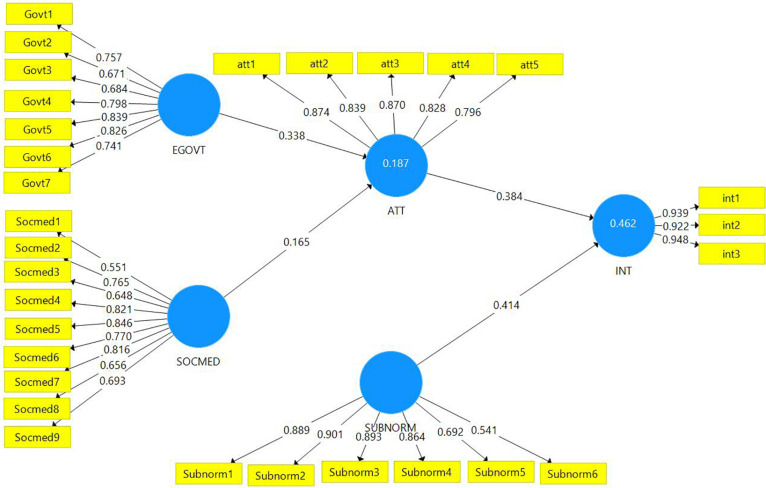
Path coefficients and structural model.

In terms of intention to adopt preventive behavior, attitude toward preventive behavior (β = 0.383, *p* < 0.001) and subjective norm (β = 0.414, *p* < 0.001) were significant antecedents. In terms of attitude toward preventive behavior, perception of e-government information and services (β = 0.338, *p* < 0.001) and perception of social media (β = 0.165, *p* < 0.001) were significant antecedents. Thus, all main effect hypotheses (i.e., H1, H2, H3, and H4) were supported. Figure 2 demonstrates the path coefficients and structural model.

The mediating effects of attitude toward preventive behavior on the intention to adopt preventive behavior were examined. It was found that an indirect effect exists between perception of e-government information and services and intention toward preventive behavior through attitude toward preventive behavior, supporting H5 (β = 0.130, *p* < 0.001). Similarly, an indirect effect also exists between perception of social media and intention to adopt preventive behavior through attitude toward preventive behavior, supporting H6 (β = 0.064, *p* < 0.05). Further examinations on the findings revealed that, in both situations, full mediation effects occur because the indirect effects were significant but not the direct effects (Baron and Kenny, 1986). The mediator variable fully explains the relationships between the dependent and independent variables (Hair et al., 2017).

### Effect Size and Predictive Relevance

The model was then assessed to check the predictive capability using evaluative metrics of predictive relevance (*Q*^2^), coefficient of determination (*R*^2^), and effect size (*f*^2^) (Table 5). The value of *Q*^2^ for endogenous constructs was 0.129 for attitude toward preventive behavior and 0.392 for intention to adopt preventive behavior, which was larger than zero, indicating predictive relevance for each dependent construct. The *R*^2^ value attitude toward preventive behavior was 0.187, indicating that perception of e-government information and services and perception of social media explained 18.7% of the variance in attitude toward preventive behavior. The *R*^2^ value for intention to adopt preventive behavior was 0.462, indicating that attitude toward preventive behavior and subjective norm explained 46.2% variance in intention to adopt preventive behavior. To check the contribution of each exogenous construct in predicting the endogenous construct, Cohen’s *f*^2^ was calculated. According to Cohen (1988), the value of *f*^2^ = 0.02, 0.15, and 0.35 demonstrated as weak, moderate, and strong effect size, respectively. The *f*^2^ value ranged from 0.028 and 0.254, indicating weak and moderate effect sizes.

**TABLE 5:** Effect size and predictive relevance.

Endogenous variables	*Q*^2^	*R*^2^	Exogenous variables	*f*^2^
ATT	0.129	0.187	EGOVT	0.117
			SOCMED	0.028
INT	0.392	0.462	ATT	0.218
			SUBNORM	0.254

*EGOVT, perception of e-government information and services; SOCMED, perception of social media; SUBNORM, subjective norm; ATT, attitude toward preventive behavior; INT, intention to adopt preventive behavior.*

## Discussion

As the COVID-19 pandemic evolves, public engagement in preventive behavior is one of the important measures that need to be improved to contain the transmission of the coronavirus. It is thus increasingly important to understand what drives people to adhere to preventive behavior as recommended by the public health authorities. Using TRA as the underpinning theory, this study empirically tested the drivers that influence people’s intention to adopt preventive behavior. The research results indicated that the proposed model could comprehensively explain the predicted behavior.

This research has demonstrated that attitude toward preventive behavior and subjective norm significantly affect intention to adopt preventive behavior. The direct effects of attitude and subjective norm on behavioral intention are consistent with previous studies (Ramayah et al., 2013; Register-Mihalik et al., 2013; Chang and Chen, 2014; Lujja et al., 2016; Dakduk et al., 2017; Kim et al., 2020). From the findings, it can be concluded that stimulating a positive attitude and social pressure could improve an individual’s intention to adopt preventive behavior. Compared to attitude, the beta value revealed that subjective norm was a more powerful driver in predicting intention to adopt preventive behavior. This signifies that interventions aimed at promoting preventive behavior should highlight the obligation to people that are important to them (Hagger et al., 2020). The findings also revealed that attitude is a reliable mediator. This proves that the effect of perception of e-government information and services and perception of social media on the intention to adopt preventive behavior during a pandemic can be fully mediated by people’s attitudes.

This study found that perception of the information and services provided over the e-government platforms was associated with attitude toward preventive behavior. The more positively the participants viewed the information and services provided over e-government, the more positive their attitude toward taking preventive actions. Perception has been associated with the development of an attitude, which in turn influences behavior (Pickens, 2005). This finding shed some light on the importance of developing a positive perception of e-government implementation among the citizen during a pandemic by providing accurate and reliable information and services. As people were isolated and limit their movements, the government should leverage the use of ICTs for public communication to ensure that they receive the right information about protecting themselves from the virus. In addition, the delivery of digital services enables the citizens to continue receiving essential government services. This implies that the commitment shown by the government to provide information and services through e-government platforms gives a positive perception to the public, which then improves their attitudes toward the government’s recommendation of preventive behavior adoption (Dai et al., 2020).

Another factor affecting attitude toward preventive behavior is the perception of social media. The more positively the participants viewed the social media, the more positive their attitude toward preventive behavior adoption. Past studies have explored the link between social media and public health (Laranjo et al., 2014; Smailhodzic et al., 2016; Sharma et al., 2017; Misra et al., 2018). The finding of the current study stresses the importance of creating positive perceptions of social media during a pandemic. This could be done by ensuring that the information shared on social media is reliable. Merchant and Lurie (2020) asserted that social media can be an efficient and user-friendly tool for health surveillance if the utilization can be more accurate and scientific.

Overall, this study provides a better understanding of how individuals’ intention to adopt preventive behavior could be influenced by attitude and subjective norms and suggests the potential positive role of e-government and social media channels and platforms in helping people decide to take preventive actions.

### Theoretical and Practical Implications

This study presents several theoretical implications, particularly on applying the TRA in public health promotion during the COVID-19 pandemic. First, the impacts of perception of e-government information and services and perception of social media on attitude toward preventive behavior were examined for the first time in this study. The findings add to the growing literature on the importance of online platforms in pandemic outbreak management. Second, the results validated the mediating effect of attitude toward preventive behavior on the relationship between the independent variables, perception of e-government information and services and perception of social media, and the dependent variable, intention to adopt preventive behavior, which is another contribution to the literature.

The research findings also provide practical implications for government health officials and decision-makers, particularly in the public health promotion during the COVID-19 pandemic. The article provides insights on the factors that affect the future adoption of preventive behavior. It helps the disease management team on how to orient public communication efforts to improve preventive behavior adoption by promoting favorable attitudes toward the behavior. Preventive behavior adoption may be increased through the usage of the subjective norm. Malaysia is a collectivistic society where members of the group are valued, and an individual’s decision to perform a specific behavior is influenced by people that are important to him/her. Therefore, public health authorities may enhance the sense of community in public health communication and promotion to encourage preventive behavior adoption.

This research supports the applications of e-government and social media in Malaysia for health communication. For a developing country like Malaysia, which have limited resources and surveillance system to manage infectious disease outbreak, e-government and social media could be the right tools to control the spread of COVID-19. With the internet accessibility, e-government and social media could provide quick and accurate information related to the disease within the communities. Therefore, government and health authorities should pay more attention to the use of e-government and social media for information dissemination in improving people’s adherence to public health recommendations during the COVID-19 outbreak.

### Limitations and Directions for Future Research

The first limitation pertains to data collection. The data were collected from Malaysian residents using a convenience sampling method. Therefore, the sample cannot be considered representative of the Malaysian population. Moreover, the majority of the participants were young, and this may lead to bias toward higher social media usage. A future study using probability sampling and including more samples in the underrepresented age group is warranted. Second, the data were self-reported and subject to bias or inaccurate reporting, which may introduce measurement error and affect the research model. Moreover, self-reported data were also at risk of the tendency to report socially desirable responses. This is because adopting preventive behavior during a pandemic is likely to be considered desirable, and respondents might be inclined to give positive responses. Future studies may advance the data collection method by mixing it with other methods that do not rely on self-report data, such as observation. Third, this study assessed the participants’ perception of e-government and social media. It is desirable to extend the study by evaluating the actual usage to get a better understanding of the effectiveness of these two platforms in mitigating the spread of COVID-19. This should include the usage frequency, the type of information that people get exposed to on these channels, and its impact on attitude, intention, and behavior. Finally, there are various social media channels with different information flowing over it. Future research should examine the relationship between social media channel that people use with the type of information they received, as different types of information will influence people’s attitudes and behavior differently.

## Conclusion

The present study aimed to identify drivers of intention to adopt preventive behavior against COVID-19 transmission. The theoretical framework was developed based on the theory of reasoned action. The results provide adequate support for the proposed model, highlighting the perception of e-government information and services and the perception of social media to promote positive attitude toward preventive behavior adoption. Improving public engagement in preventive behavior against COVID-19 is necessary to maintain public health.

## Data Availability Statement

The raw data supporting the conclusions of this article will be made available by the authors, without undue reservation.

## Ethics Statement

The studies involving human participants were reviewed and approved by the Monash University Human Research Ethics Committee (Project ID: 24906). The participants provided their implied informed consent to participate in this study.

## Author Contributions

NM conceptualized the idea, collected and analyzed the data, and writing the original manuscript. HN contributed in data collection and data analysis, and edited the original draft. PM contributed in data analysis and edited the revised manuscript. All authors contributed to the article and approved the submitted version.

## Conflict of Interest

The authors declare that the research was conducted in the absence of any commercial or financial relationships that could be construed as a potential conflict of interest.

## Footnotes

1 covid-19.moh.gov.my
